# Identification of Ly2 members as antimicrobial peptides from zebrafish *Danio rerio*

**DOI:** 10.1042/BSR20160265

**Published:** 2017-01-17

**Authors:** Xuemin Liu, Xuwen Cao, Su Wang, Guangdong Ji, Shicui Zhang, Hongyan Li

**Affiliations:** 1 Department of Marine Biology, Institute of Evolution and Marine Biodiversity, Ocean University of China, Qingdao 266003, China; 2 Department of Marine Biology, Laboratory for Evolution and Development, Ocean University of China, Qingdao 266003, China

**Keywords:** zebrafish Danio rerio, Ly6 gene cluster, Antimicrobial peptides

## Abstract

The emergence of multidrug-resistant (MDR) microbes caused by overuse of antibiotics leads to urgent demands for novel antibiotics exploration. Our recent data showed that Ly2.1–3 (a novel lymphocyte antigen 6 (*Ly6*) gene cluster) were proteins with cationic nature and rich in cysteine content, that are characteristic of antimicrobial peptides (AMPs) and their expression were all significantly up-regulated after challenge with lipopolysaccharide (LPS). These strongly suggested that Ly2.1–3 are potential AMPs, but firm evidence are lacking. Here, we clearly showed that the recombinant proteins of Ly2.1–3 were capable of killing Gram-negative bacteria *Aeromonas hydrophila* and *Escherichia coli*, while they had little bactericidal activity against the Gram-positive bacteria *Staphylococcus aureus* and *Bacillus subtilis*. We also showed that recombinant proteins Ly2.1–3 (rLy2.1–3) were able to bind to the Gram-negative bacteria * A. hydrophila,* *E. coli* and the microbial signature molecule LPS, but not to the Gram-positive bacteria * S. aureus* and *B. subtilis* as well as the microbial signature molecule LTA. Moreover, the Scatchard analysis revealed that rLy2.1–3 could specifically bind to LPS. Finally, we found that Ly2.1–3 were not cytotoxic to mammalian cells. All these together indicate that Ly2.1–3 can function as AMPs.

## Introduction

Antimicrobial peptides (AMPs), which are found in all living organisms ranging from bacteria to humans, are an evolutionarily conserved component of the innate immune system [1,2]. AMPs exhibit a broad spectrum of activity against bacteria, fungi, yeasts, protozoa and viruses. Besides their antimicrobial activity, some AMPs are also recognized for their immunomodulatory properties [3]. AMPs are typically <100 amino acids in length, display hydrophobic and cationic properties and adopt an amphipathic structure [2,4]. Generally, the antibacterial peptide contains rich cysteine residues and forms disulfide bonds. The global ever-growing concerns about multidrug-resistant (MDR) microbes lead to urgent demands for exploration of new antibiotics including AMPs. To date, more than 2400 AMPs have been reported in the Antimicrobial Peptide Database (http://aps.unmc.edu/AP/main.php), and they exhibit tremendous sequence diversity.

Lymphocyte antigen 6 (Ly6) is a superfamily of proteins that includes a conserved LU domain with eight or ten cysteine residues, forming four to five conserved disulfide bonds. The biological functions of Ly6 superfamily members are diverse, and our knowledge as such has been increasingly accumulated. For example, in mammals, Ly6 proteins such as E48, ly6A.2, ly6C and urokinase-type plasminogen activator receptor (uPAR) have been shown to play roles in cell adhesion and migration through interaction with specific receptors [5–8], lynx1 have been shown to regulate the activity of nicotinic acetylcholine receptors [9,10] and lynx2 have been shown to correlate with anxiety-related behaviour via neuronal nicotinic receptors [11]. Some Ly6 members are also associated with diseases. PSCA has been shown to be overexpressed in prostate cancer [12,13], and SLURP1 is absent in Mal de Meleda [14]. In spite of these progresses, functions of most Ly6 members remain ill-defined.

Recently, it has been shown that zebrafish CD59, a member of Ly6 superfamily, is an immune-relevant molecule capable of binding to and inhibiting the growth of bacteria including *Escherichia coli* and *Staphylococcus aureus* [15]. In addition, we have previously showed that another three members of Ly6 superfamily, Ly2.1–3 (Ly2.1, Ly2.2 and Ly2.3), were highly identical with each other at amino acid levels [16]. Sequence alignment of LU domains revealed that many of the secreted proteins including Ly2.1–3 and the secreted members of Ly6 SF SLURP1, SLURP2, typical PATE proteins and TFTs share low homology, except the conserved cysteine residues. The predicted Ly2.1–3 proteins all contained a signal peptide and single LU domain with ten position-specific conserved cysteines forming five disulfide bonds. They code for three secreted proteins consisting of 91, 95 and 94 amino acids with predicted molecular weights of approximately 10.02, 10.29, 10.29 kDa respectively [16]. Ly2.1–3 are proteins with cationic nature and rich in cysteine content, that are characteristic of AMPs. Ly2.1–3 exhibit distinct expression pattern in the endoderm, they were found abundantly and specifically in the digestive tract, liver and pancreas respectively. And the digestive system of fish plays a key role in innate immunity and adaptive immunity to defend against pathogens [17–19]. Furthermore, challenge with lipopolysaccharide (LPS) resulted in a marked increase in the expression of *Ly2.1*, *Ly2.2* and *Ly2.3* genes. These strongly suggest that Ly2.1, Ly2.2 and Ly2.3 are AMPs [16]. However, firm evidence supporting this suggestion remains lacking. The aims of the present study are thus to examine if Ly2.1, Ly2.2 and Ly2.3 have antibacterial activity, and if so, to explore their modes of action.

## Materials and methods

### Expression and purification of rLy2.1–3 and TRX-His-tag peptide

The cDNA regions encoding the mature peptides of Ly2.1–3 were amplified by PCR with the specific primers rS1 and rAS1, rS2 and rAS2, rS3 and rAS3 respectively (Supplementary Table S1). The reaction was performed under the following conditions: an initial denaturation at 94°C for 5 min, followed by 35 cycles at 94°C for 30 s, 55°C for 30 s and 72°C for 30 s and a final extension at 72°C for 7 min. The PCR products were each digested with EcoRI and XhoI, and subcloned into the plasmid expression vector pET28a (Novagen) previously cut with the same restriction enzymes. The identity of the inserts was verified by sequencing, and the plasmids were designated as pET28a/Ly2.1, pET28a/Ly2.2 and pET28a/Ly2.3 respectively.

The cells of *E. coli* Transetta (DE3) were transformed with the constructed plasmids pET28a/Ly2.1, pET28a/Ly2.2 or pET28a/Ly2.3, and then cultured overnight in LB broth containing 50 μg/ml kanamycin. The cultures were each diluted to 1:1000 with LB broth and incubated at 37°C for 6 h. The production of Ly2.1, Ly2.2 and Ly2.3 was auto-induced by the protein expression system [20]. The auto-induction was continued at 37°C overnight, then *E. coli* cells were collected by centrifugation, resuspended in ice-cold lysis buffer (50 mM Tris, 100 mM NaCl and 1 mM EDTA, pH 6.5) and sonicated on ice. After centrifugation at 12000 ***g*** at 4°C for 20 min, the pellets consisting of the inclusion bodies were pooled and washed with washing buffer (50 mM Tris, 100 mM NaCl, 0.5 mM EDTA and 2 M urea, pH 6.5). After solubilization in dissolving buffer (100 mM Tris, 500 mM NaCl, 10 mM imidazole and 8 M urea, pH 6.5), the recombinant proteins Ly2.1–3 (rLy2.1–3) were purified by Ni-nitrilotriacetic acid resin column. The purity of the recombinant proteins was analysed by SDS/PAGE (12% gel) and stained with Coomassie Brilliant Blue R-250.

To express the TRX-His-tag peptide as control, DE3 cells were also transformed by plasmid pET-32a(+) (Novagen) and induced with IPTG at a final concentration of 0.5 mM at 28°C for 8 h. The peptide was purified and proceeded as described above.

### Refolding of rLy2.1-3

The purified rLy2.1-3 were dialysed successively in the dialysis buffer I (25 mM Tris, 200 mM NaCl, 1 mM EDTA, 5 M urea and 5 mM DTT, pH 6.5) for 6 h; dialysis buffer II (25 mM Tris, 50 mM NaCl, 1 mM EDTA, 4 M urea, 0.2 mM GSSG and 2 mM GSH, pH 6.5) for 6 h; dialysis buffer III (25 mM Tris, 20 mM NaCl, 1 mM EDTA, 2 M urea, 0.5 mM GSSG and 1 mM GSH, pH 6.5) for 6 h; dialysis buffer IV (25 mM Tris, 10 mM NaCl, 1 mM EDTA, 1 M urea, 5% glycerol, 5 mM DTT, 1 mM GSSG and 1 mM GSH, pH 6.5) for 6 h; dialysis buffer V (25 mM Tris, 10 mM NaCl, 1 mM EDTA, 0.5 M urea, 5% (v/v) glycerol, 1% (w/v) glycine, 5 mM DTT, 1 mM GSSG, 1 mM GSH, pH 6.5) for 6 h; dialysis buffer VI (25 mM Tris, 10 mM NaCl, 5% glycerol, 1% (w/v) glycine and 5 mM DTT, pH 6.5) for 6 h; and dialysis buffer VII (25 mM Tris and 10 mM NaCl, pH 6.5) for 6 h. The protein concentrations were determined by the method of BCA using BSA as a standard.

### Western blotting

The purified recombinant proteins were run on an SDS/PAGE (12% gel). The proteins on the gel were electroblotted on to PVDF membrane (Amersham) by a semi-dry technique. The blotted membranes were blocked with 4% BSA in 10 mM PBS (pH 7.4) at room temperature for 3 h, and then incubated with the mouse anti-His-tag antibody (CWBIO) diluted to 1:5000 with 10 mM PBS (pH 7.4) at 4°C overnight. After washing in 10 mM PBS (pH 7.4) containing 0.1% Tween-20, the membranes were incubated with horseradish peroxidase (HRP)-labelled goat anti-mouse IgG (CWBIO) diluted to 1:8000 at room temperature for 1 h. The bands were visualized using DAB.

### Antimicrobial activity assay

A colony formation assay was used to test the bactericidal activity of Ly2.1–3 against *Aeromonas hydrophila*, *E. coli*, *S. aureus* and *Bacillus subtilis* as described by Milochau et al. [21]. In brief, the strains of *A. hydrophila*, *E. coli*, *S. aureus* and *B. subtilis* were cultured in TSB and LB media respectively. They were cultured to mid-exponential phase, and then collected by centrifugation at 6000 ***g*** at room temperature for 5 min. The bacterial pellets were washed in 25 mM Tris buffer containing 10 mM NaCl or TBS three times, resuspended in Tris/NaCl buffer or TBS and adjusted to a density of 3 × 10^4^ cells/ml. Totally, 50 μl of *A. hydrophila*, *E. coli*, *S. aureus* and *B. subtilis* suspensions was each pre-incubated at 25°C for 1 h with 50 μl of recombinant Ly2.1, Ly2.2 and Ly2.3, TRX-His-tag peptide (negative control) or ampicillin (positive control) (50, 100 and 200 μg/ml) solutions, yielding final concentrations of 25, 50 and 100 μg/ml respectively, and then each of these bacterial suspensions was plated on to three agar plates (30 μl/plate). After incubation at 37°C for 12 h, the resulting bacterial colonies in each plate were counted. The percentage of bactericidal activity was calculated as follows: [number of colonies (control − test)/number of colonies (control)] ×100% (*n*=3). The control was processed similarly except that Tris/NaCl buffer or TBS was used instead of protein solution.

### Assay for binding of recombinant proteins to bacteria

The strains of *A. hydrophila*, *E. coli*, *S. aureus* and *B. subtilis* were cultured in TSB and LB media respectively. They were cultured to mid-exponential phase, and then they were collected by centrifugation at 6000 ***g*** at room temperature for 5 min. The bacterial pellets were resuspended in 25 mM Tris buffer containing 10 mM NaCl or TBS (pH 6.5), giving a concentration of 1 × 10^8^ cells/ml and used for the following experiments.

Aliquots of 150 μl of *A. hydrophila*, *E. coli*, *S. aureus* and *B. subtilis* suspension (1 × 10^8^ cells/ml) were mixed with 300 μl of 100 μg/ml rLy2.1, rLy2.2, rLy2.3 or TRX-His-tag peptide in 25 mM Tris buffer with 10 mM NaCl (pH 6.5) or with the same volume of Tris/NaCl solution alone. The mixtures were incubated at 25°C for 2 h, and the bacteria were then collected by centrifugation at 6000 ***g*** at room temperature for 5 min. After washing in Tris/NaCl solution, the bacterial pellets were resuspended in Tris/NaCl solution (pH 6.5), giving a concentration of 2 × 10^7^ cells/ml. Both recombinant protein-treated bacteria and non-treated bacteria as well as purified rLy2.1–3 or TRX-His-tag peptide were run on an SDS/PAGE (12% gel), followed by Western blotting analysis using anti-His tag antibody as described above.

### Assay for binding of Ly2.1–3 to LPS and LTA

Both rLy2.1–3 and TRX-His-tag peptide were individually biotinylated with biotinamidohexanoic acid *N*-hydroxysuccinimide ester (NHS-LC-biotin; HEOWNS, Tianjin, China) as described by Zhang et al. [22]. Briefly, 100 μl of 2 mg/ml NHS-LC-biotin was mixed with 1 ml of Ly2.1, Ly2.2 or Ly2.3 (0.1 mg/ml) or TRX-His-tag peptide (0.1 mg/ml) solution, incubated at 25°C for 2 h and then 20 μl of 0.5 M (NH_4_)_2_SO_4_ was added to the mixture to inactivate the uncombined biotin. The uncombined biotin was removed by ultrafiltration. To test the binding of Ly2.1–3 to LPS and LTA, 50 μl of 40 μg/ml LPS or LTA in 10 mM PBS (pH 7.4) was applied to each well of a 96-well microplate and air-dried at 25°C overnight. The plate was incubated at 60°C for 30 min to further fix LPS or LTA, and the wells were each blocked with 200 μl of 40 mg/ml BSA in 10 mM PBS (pH 7.4) at 37°C for 2 h. After washing four times with 200 μl of 10 mM PBS supplemented with 0.5% Tween-20, 50 μl of biotinylated Ly2.1–3 (0, 2, 4, 6, 8, 10, 12, 15, 20 and 25 μg/ml) was added to each well. After incubation at room temperature for 3 h , the wells were each rinsed four times with 200 μl of 10 mM PBS supplemented with 0.5% Tween-20 and 100 μl of streptavidin–HRP (CWBIO) diluted to 1:2000 with 10 mM PBS (pH 7.4) containing 1 μg/ml BSA was added to each well. After incubation at room temperature for 1 h, the wells were washed four times with 200 μl of 10 mM PBS supplemented with 0.5% Tween-20, added with 75 μl of 0.4 mg/ml *O*-phenylenediamine (Amresco) in the buffer consisting of 51.4 mM Na_2_HPO_4_, 24.3 mM citric acid and 0.045% H_2_O_2_ (pH 5.0) and reacted at 37°C for 5 to 30 min. Subsequently, 25 μl of 2 M H_2_SO_4_ was added to each well to terminate the reaction and absorbance at 492 nm was monitored by a microplate reader. For control, recombinant TRX-His-tag peptide at the same concentrations was treated similarly.

For Scatchard analysis, LPS and LTA were individually biotinylated with NHS-LC-biotin (Heowns, Tianjin, China) as described by Zhang et al. [22]. Fifty microlitres of 40 μg/ml rLy2.1–3 in 10 mM PBS (pH 7.4) were applied to each well of a 96-well microplate and air-dried at 25°C overnight. The plate was incubated at 60°C for 30 min to further fix rLy2.1–3, and the wells were each blocked with 200 μl of 40 mg/ml BSA in 10 mM PBS (pH 7.4) at 37°C for 2 h. After washing four times with 200 μl of 10 mM PBS supplemented with 0.5% Tween-20, 50 μl of biotinylated LPS or LTA (0, 2, 4, 6, 8, 10, 20, 30, 40 and 60 μg/ml) was added to each well and then processed as above. For control, recombinant TRX-His-tag peptide instead of rLy2.1–3 at the same concentrations was treated similarly. The equilibrium dissociation constant (*K*_d_) and apparent maximum number of bindings (*B*_max_) were determined according to the Scatchard plot using the software GraphPad Prism 5.01.

### MTT assay

To test if Ly2.1–3 are cytotoxic to murine RAW264.7 cells, MTT assay was performed as described by Hu et al. [23]. RAW264.7 cells were suspended in serum-free DMEM and aliquots of 180 μl of the cell suspension (1 × 10^6^ cells/ml) were sampled into a 96-well plate and cultured at 37°C under 5% CO_2_ for 2 h. Subsequently, aliquots of 200 μl of Ly2.1–3 in serum-free DMEM were added to each well, giving final concentrations of 25, 50, 100 and 150 μg/ml respectively, incubated for another 4 h, and then 20 μl of MTT solution (5 mg/ml in PBS) was added into each well. After incubation for 4 h, the medium was removed and 150 μl of DMSO were added. The absorbance at 492 nm was measured under a microplate reader. The cells treated with DMEM alone served as control. The percent of viability against the control was calculated as follows: (*A* of treated groups/*A* of control groups) × 100% (*n*=3).

### Haemolytic activity assay

Human red blood cells (RBCs) were used to test the haemolytic activity of Ly2.1–3 as described by Hu et al. [23]. Healthy human blood was obtained in an EDTA anticoagulant tube, and RBCs were collected by centrifugation at room temperature at 1000 ***g*** for 10 min. After washing three times with PBS (pH 7.4), RBCs were suspended in PBS to give a concentration of 4% (v/v). An aliquot of 200 μl RBCs suspension was mixed with 200 μl of Ly2.1–3 solution, giving final concentrations of 25, 50, 100 and 150 μg/ml. After incubation at 37°C for 1 h, the mixtures were centrifuged at room temperature at 1000 ***g*** for 10 min. The supernatants were collected and added into a 96-well plate. The absorbance was measured at 540 nm under a microplate reader (Multiskan GO, Thermo Scientific). RBCs incubated with PBS alone, BSA solution (150 μg/ml), 0.1% Triton X-100 solution served as blank, negative and positive controls respectively. Each treatment was performed in triplicate.

### Statistical analysis

All the experiments were conducted at least three times. Statistical analyses were performed using the GraphPad Prism 5. The significance of difference was determined by one-way ANOVA, and the difference at *P*<0.05 was considered significant. All the data were expressed as mean ± S.E.M.

## Results

### Expression and purification of rLy2.1–3 and TRX-His-tag peptide

**Figure 1:**
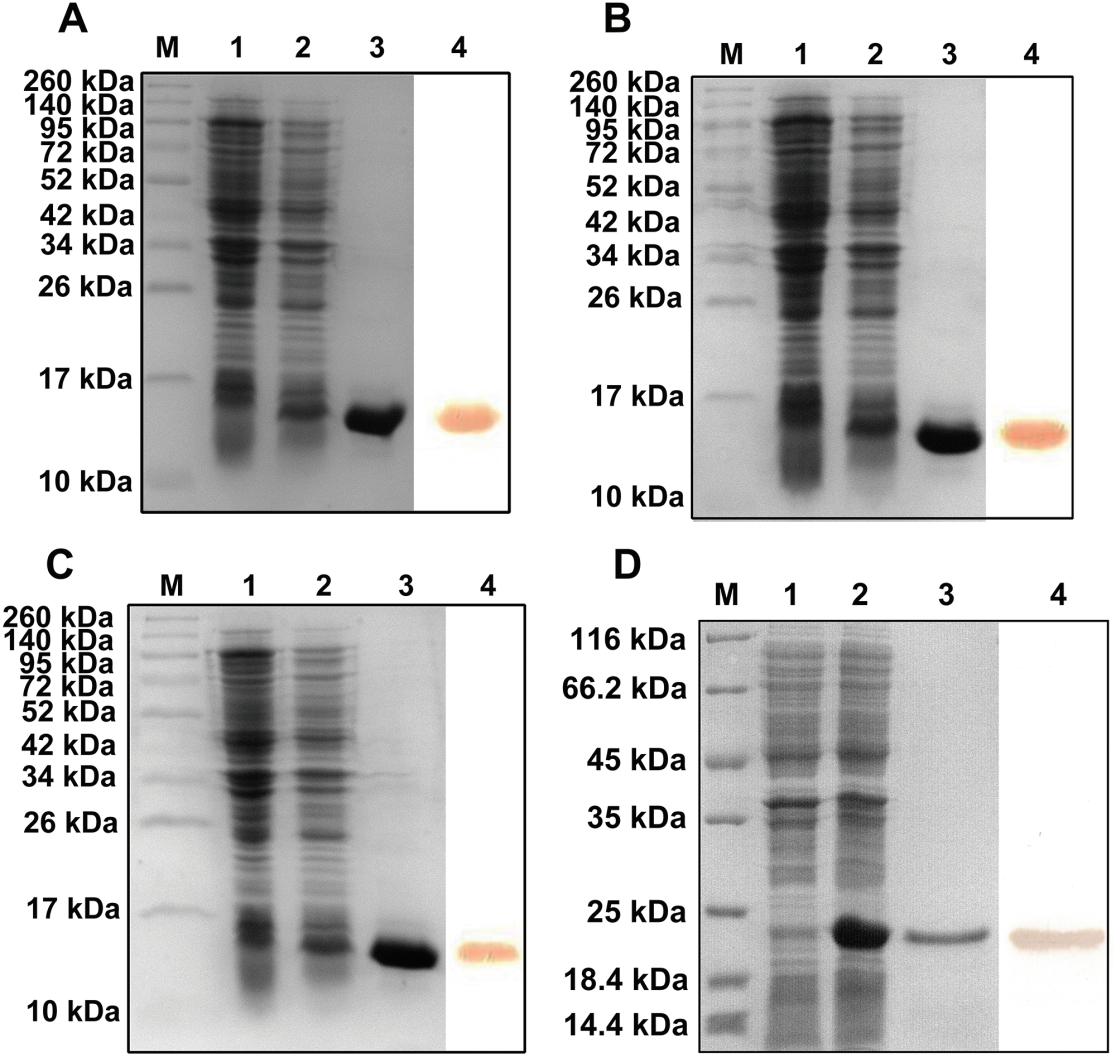
SDS/PAGE and Western blotting. SDS/PAGE and Western blotting of rLy2.1 (**A**); rLy2.2 (**B**); rLy2.3 (**C**) and TRX-His-tag peptide (**D**). Lane M: molecular mass standards; lane 1: total cellular extracts from DE3 cells containing expression vector before induction; lane 2: total cellular extracts from DE3 containing expression vector after induction; lane 3: purified recombinant proteins and lane 4: Western blot of purified recombinant proteins.

The rLy2.1–3 and TRX-His-tag peptide with the His-tag were expressed in *E. coli* and purified by affinity chromatography on a Ni-nitrilotriacetic acid resin column. The purified rLy2.1–3 and TRX-His-tag peptide all yielded a single band of approximately 12, 12, 12 and 21 kDa respectively, on SDS/PAGE gel after Coomassie blue staining, matching the expected sizes (Figure 1). Western blotting showed that rLy2.1–3 and TRX-His-tag peptide were reacted with mouse anti-His-tag antibody, indicating that rLy2.1, rLy2.2, rLy2.3 and TRX-His-tag peptide were correctly expressed (Figure 1).

### rLy2.1–3 are bactericidal agents

**Figure 2:**
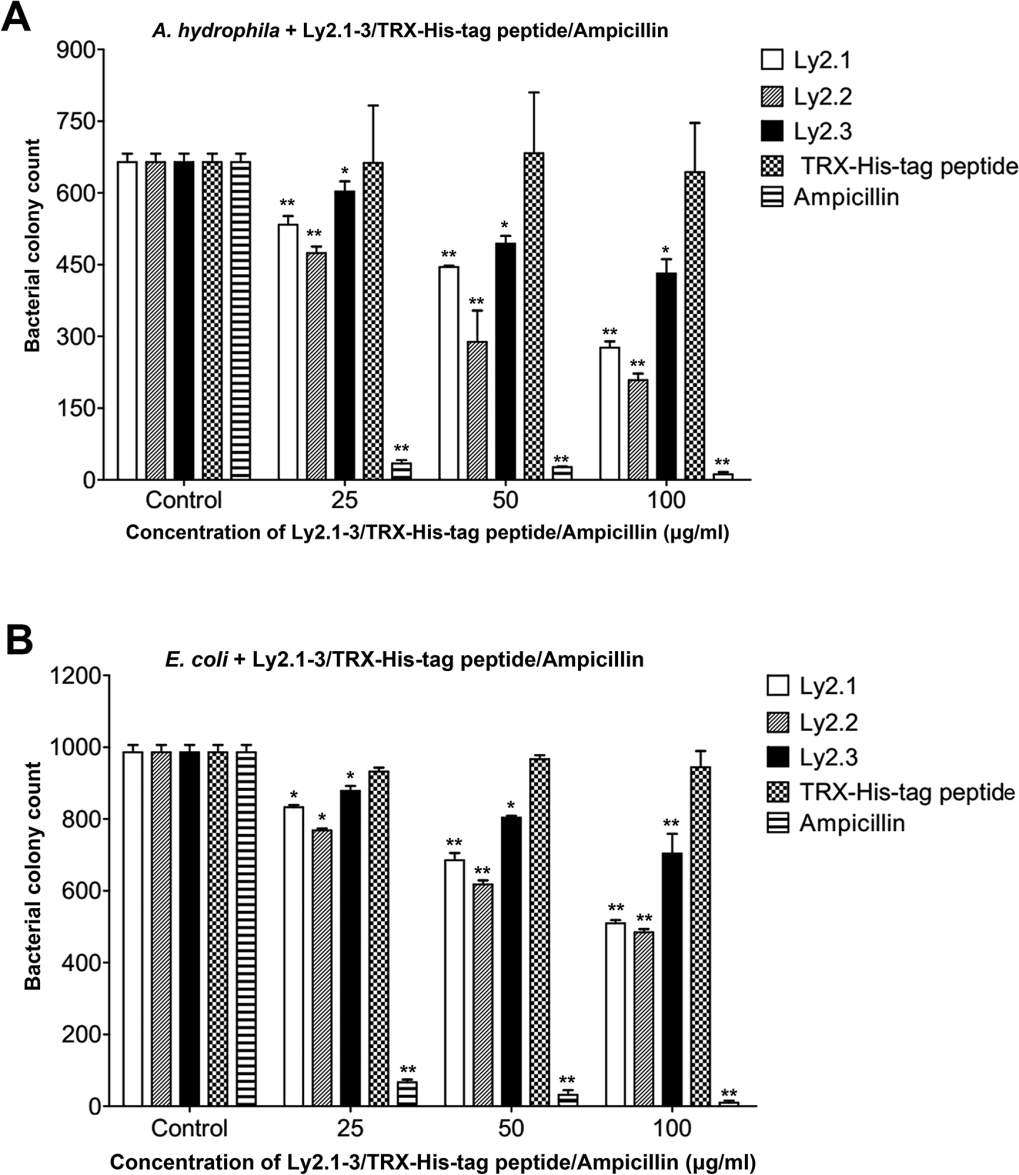
Antibacterial activity of Ly2.1-3. Antibacterial activity of Ly2.1–3 against *A. hydrophila* (**A**) and *E. coli* (**B**). (A) *A. hydrophila* incubated in the absence of rLy2.1–3/TRX-His-tag peptide was used as a control. (B) *E. coli* incubated in the absence of rLy2.1–3/TRX-His-tag peptide was used as control. Data were expressed as mean ± S.E.M. (*n*=3). The bars represent the S.E.M. values. The symbols (**) and (*) indicate *P*<0.0001 and *P*<0.001 respectively, compared with the control group.

The colony formation assay was used to test if rLy2.1–3 showed any antibacterial activity against the Gram-negative bacteria * A. hydrophila* and *E. coli*, and the Gram-positive bacteria *S. aureus* and *B. subtilis*. It was found that rLy2.1–3 were each capable of killing the Gram-negative bacteria *A. hydrophila* and *E. coli* in a dose-dependent fashion (Figure 2A and 2B, and Supplementary Figures S2 and S3). The percentage of bactericidal activity are presented in Supplementary Table S2. Compared with rLy2.3, rLy2.1 and rLy2.2 exhibited higher bactericidal activity. Additionally, rLy2.1–3 showed higher bactericidal activities against *A. hydrophila* than that of *E. coli*. By contrast, they had little bactericidal activity against the Gram-positive bacteria * S. aureus* and *B. subtilis* (Supplementary Figures S1A, S1B, S4 and S5). These indicated that rLy2.1, rLy2.2 and rLy2.3 were all able to specifically kill Gram-negative bacteria such as *A. hydrophila* and *E. coli* rather than Gram-positive bacteria. For control, recombinant TRX-His-tag peptide had no bactericidal activity against the Gram-negative bacteria *A. hydrophila* and *E. coli* and the Gram-positive bacteria * S. aureus* and *B. subtilis*. By contrast, the bactericidal activity of standard antibiotic is very strong, the bactericidal rate can reach almost 100%.

### rLy2.1–3 bind to Gram-negative bacteria

**Figure 3:**
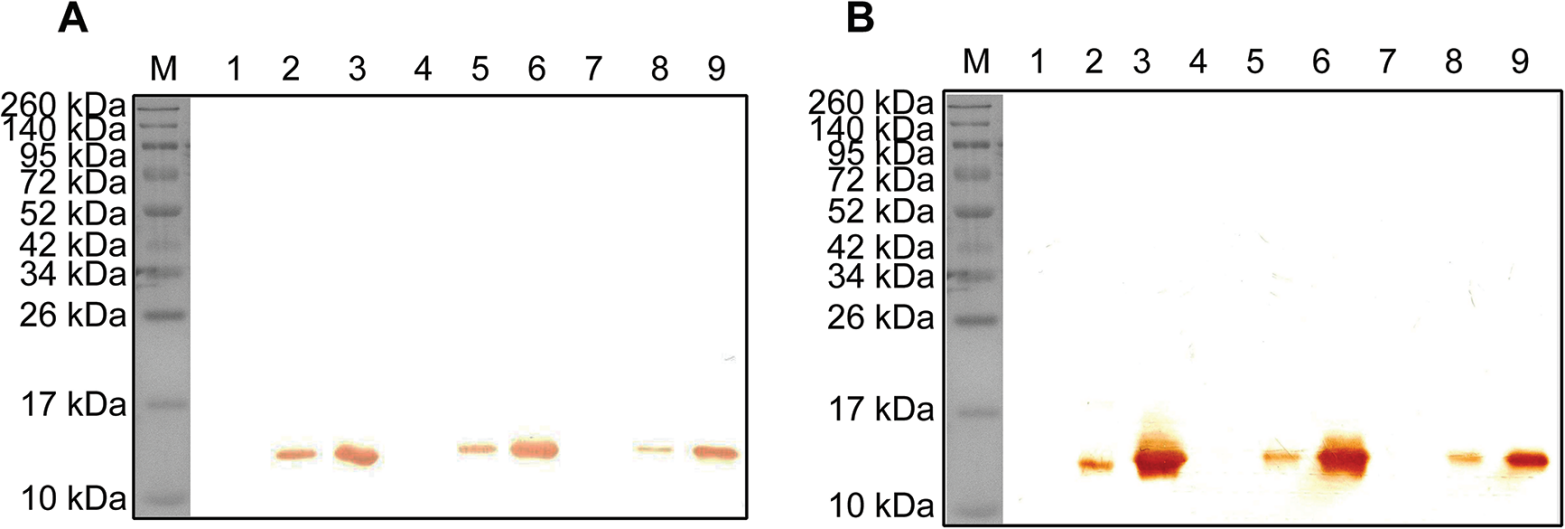
Interaction of Ly2.1-3 with microbes. Interaction of rLy2.1-3 with *A. hydrophila* (A) and *E. coli* (B). (**A**) Western blotting. Lane M: molecular mass standards; lanes 1, 4 and 7: *A. hydrophila* incubated in the absence of rLy2.1–3; lane 2: *A. hydrophila* incubated in the presence of rLy2.1; lane 3: purified rLy2.1 only; lane 5: *A. hydrophila* incubated in the presence of rLy2.2; lane 6: purified rLy2.2 only; lane 8: *A. hydrophila* incubated in the presence of rLy2.3; lane 9: purified rLy2.3 only. (**B**) Western blotting. Lane M: molecular mass standards; lanes 1, 4 and 7: *E. coli* incubated in the absence of rLy2.1–3; lane 2: *E. coli* incubated in the presence of rLy2.1; lane 3: purified rLy2.1 only; lane 5: *E. coli* incubated in the presence of rLy2.2; lane 6: purified rLy2.2 only; lane 8: *E. coli* incubated in the presence of rLy2.3; lane 9: purified rLy2.3 only.

Next, we tested if rLy2.1–3 bind to the Gram-negative bacteria *A. hydrophila* and *E. coli* and the Gram-positive bacteria * S. aureus* and *B. subtilis*. As revealed by Western blotting, rLy2.1–3 were all capable of binding to both *A. hydrophila* (Figure 3A) and *E. coli* (Figure 3B), suggesting the presence of interaction of rLy2.1–3 with Gram-negative bacteria. By contrast, rLy2.1–3 were not capable of binding to both *S. aureus* (Supplementary Figure S6A) and *B. subtilis* (Supplementary Figure S6B). For control, recombinant TRX-His-tag peptide were not capable of binding to *A. hydrophila*, *E. coli*, *S. aureus* and *B. subtilis* (Supplementary Figure S6C).

### rLy2.1–3 bind to LPS

**Figure 4:**
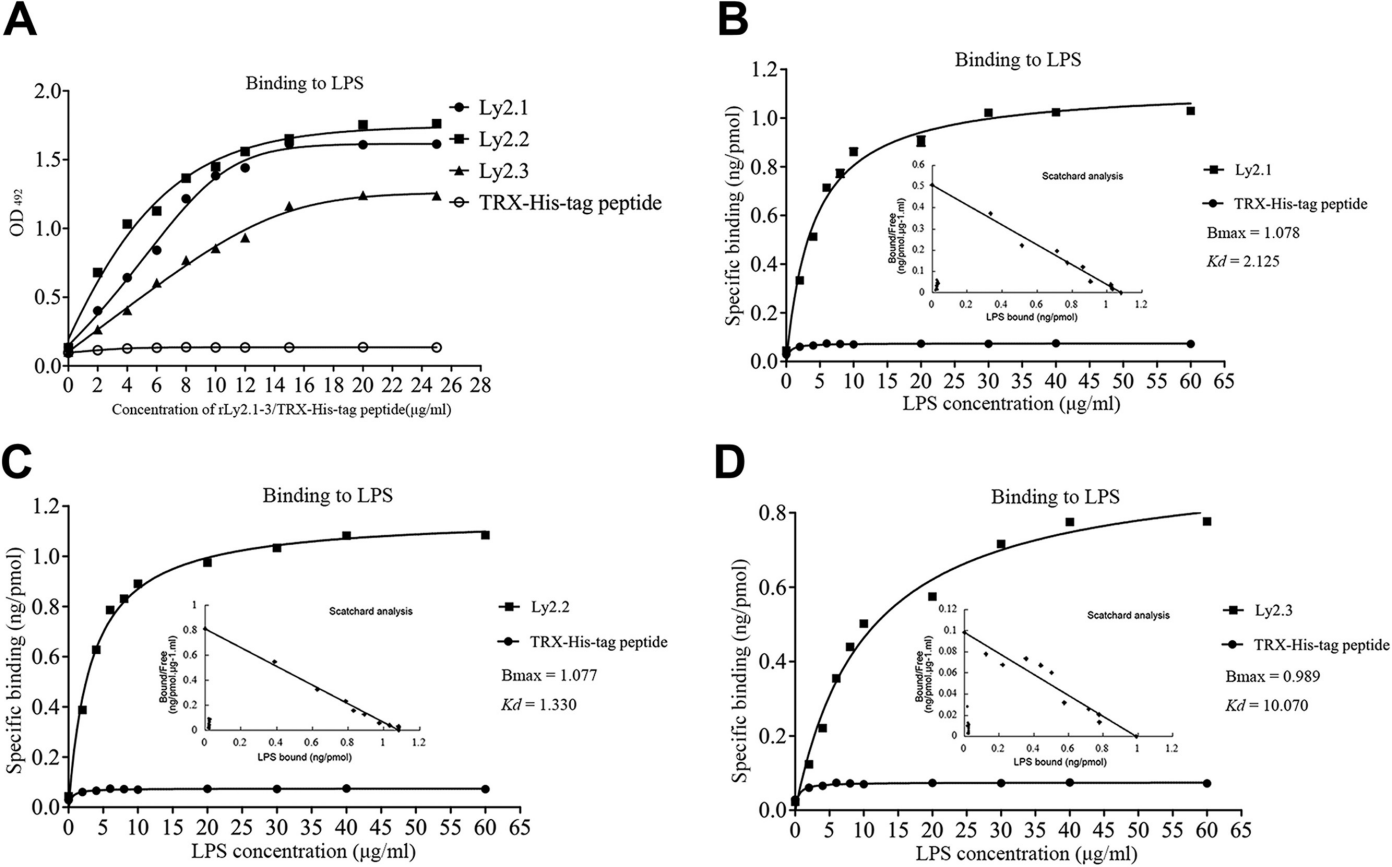
Interaction of Ly2.1-3 with microbial cell components. Interaction of rLy2.1–3 with LPS. (**A**) Binding of rLy2.1–3 to LPS. LPS dissolved in PBS (pH 7.4) was applied to wells of a 96-well microplate and air-dried overnight at room temperature, followed by ELISA. TRX-His-tag peptide instead of rLy2.1–3 was used as control. (**B**–**D**) saturation curve of the binding of Ly2.1 (B), Ly2.2 (C) and Ly2.3 (D) to LPS. rLy2.1–3 dissolved in PBS (pH 7.4) was applied to wells of a 96-well microplate and air-dried overnight at room temperature, followed by Scatchard analysis. TRX-His-tag peptide was treated at the same concentrations as control. Data were expressed as mean ± S.E.M. (*n*=3). The bars represent the S.E.M. values.

To better understand the mechanism by which rLy2.1–3 bind to the Gram-negative bacteria, ELISA was carried out to test if rLy2.1–3 bind to LPS, the signature molecule of Gram-negative bacteria. As shown in Figure 4(A), rLy2.1–3 were able to bind to LPS and the binding was dose-dependent and saturable. By contrast, recombinant TRX-His-tag peptide used as a control only showed little affinity to LPS. These data indicated that rLy2.1–3 could identify and bind to the signature molecule, LPS of Gram-negative bacteria. We also tested if rLy2.1–3 could bind to LTA, the signature molecule of Gram-positive bacteria. The data showed that rLy2.1–3 and recombinant TRX-His-tag peptide could not bind to LTA (Supplementary Figure S7). The result is in accordance with bacterial-binding assay.

To directly characterize the affinities of rLy2.1–3 to the LPS, we further performed the Scatchard analysis. The *B*_max_ (ng/pmol protein) and the *K*_d_ values (μg/ml) of the affinity of rLy2.1–3 to LPS were approximately 1.078 and 2.125, 1.077 and 1.330 as well as 0.989 and 10.070. Here, *B*_max_ value is the maximum saturation concentration of LPS bound to rLy2.1–3 (Figure 4B–4D). The results of Scatchard analysis is linear, which implies that the binding site did not play cooperatively. Therefore, the binding of rLy2.1–3 to LPS was specific and saturable. However, no specific binding of recombinant TRX-His-tag peptide to LPS was detected.

### Ly2.1–3 display little cytotoxicity

**Figure 5:**
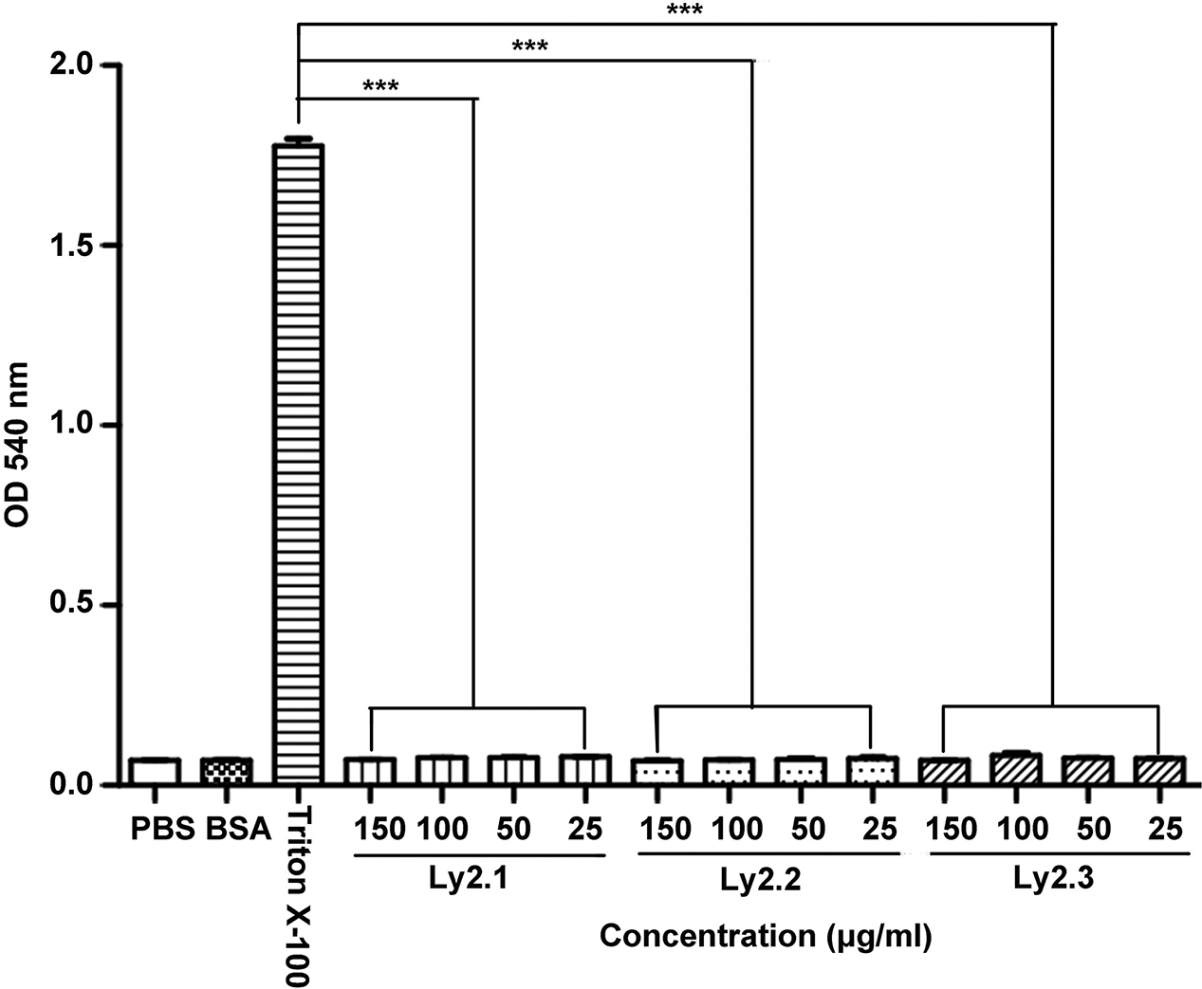
Haemolytic activity of Ly2.1-3 on human red blood cells (RBCs). Data were expressed as mean ± S.E.M. (*n*=3). The bars represent the S.E.M. values. The symbol (***) indicates *P*<0.001 compared with the Triton X-100 treated group.

To test if Ly2.1–3 were cytotoxic, their haemolytic activity on human RBCs were determined. As shown in Figure 5, Ly2.1–3 showed little haemolytic activity towards human RBCs at all the concentrations tested. By contrast, RBCs incubated with 0.1% Triton X-100 (full lysis control) exhibited significant haemolysis. In addition, the cytotoxicity of Ly2.1–3 to murine RAW264.7 cells was also tested using MTT. Ly2.1–3 were not toxic to murine RAW264.7 cells at the concentrations tested (Table 1). These data showed that Ly2.1–3 were non-cytotoxic to mammalian cells, suggesting that Ly2.1–3 have a high bacterial membrane selectivity.

**Table 1:** The percent of viability of RAW264.7 cells in the presence of Ly2.1–3

Gene	Concentration (μg/ml)	Percent of viability (%)
	0	100
	25	108 ± 4
*Ly2.1*	50	100 ± 4
	100	99 ± 4
	150	110 ± 4
	25	99 ± 3
*Ly2.2*	50	100 ± 3
	100	101 ± 5
	150	104 ± 3
	25	106 ± 3
*Ly2.3*	50	107 ± 1
	100	106 ± 5
	150	104 ± 5

## Discussion

The Ly-6 (also known as Ly-6/uPAR) superfamily of molecules was first identified in mouse, murine Ly-6-encoded molecules play an important role in the antigen-independent activation of lymphocytes [24]. And Ly-6/uPAR molecules have an interesting expression pattern during haematopoiesis indicating that Ly-6/uPAR molecules are associated with development of the immune system [25]. Recent study suggested that Ly6/PLAUR domain containing 8 (Lypd8) prevents flagellated microbiota from invading the colonic epithelia in mice [26]. Although Lypd8 exhibited no bactericidal activity against *Proteus mirabilis*, Lypd8 bound to flagella and suppressed motility of flagellated bacteria. Thus, Lypd8 mediates segregation of intestinal bacteria and epithelial cells in the colon to preserve intestinal homoeostasis. Therefore, in mammals, the members of Ly6 superfamily play a critical role in defending themselves *in vivo*.

A member of Ly6 superfamily has been identified in various metazoans, but functions of most Ly6 members remain poorly characterized. Recent study suggested that the members of Ly6 superfamily Ly2.1, Ly2.2 and Ly2.3 are potential AMPs [16]. In the present study, we clearly demonstrated that rLy2.1–3 all possess bactericidal activity against Gram-negative bacteria, confirming that they are indeed AMPs. We used two Gram-negative bacteria *A. hydrophila* and *E. coli* and two Gram-positive bacteria *S. aureus* and *B. subtilis* to test the bactericidal activity of recombinant Ly2.1–3. The data indicated that Gram-negative strains are more susceptible to Ly2.1–3 than Gram-positive strains. This specific selection is similar to that of medaka β-defensin peptide and human β-defensin 2 (HBD-2) [27,28]. In addition, compared with standard antibiotics, the bactericidal activity of recombinant Ly2.1–3 were much weaker. Among them, the bactericidal activity of recombinant Ly2.3 was the weakest. rLy2.1–3 were not only able to bind to the Gram-negative bacteria tested but also to their signature molecule LPS. To directly characterize the affinities of rLy2.1–3 to the LPS, we further performed the Scatchard analysis. It was clear that rLy2.3 showed the weakest affinity to LPS. Ly2.1 shared 79.1% identity with that of Ly2.2, while Ly2.1 and Ly2.2 shared 44.0% and 44.7% identity with that of Ly2.3. We can find that Ly2.1 and Ly2.2 share higher homology compared with Ly2.3, which may lead to the lowest affinity of Ly2.3 to LPS. We speculate that is the reason Ly2.3 is less active compared with Ly2.1 and Ly2.2. These data hint a clue that Ly2.1–3 may function as pattern recognition molecules capable of identifying the Gram-negative bacteria. Recently, Wang et al. [29] identified the zinc finger protein ZRANB2 as a novel maternal LPS-binding protein that protects embryos of zebrafish against Gram-negative bacterial infections and they also found rZRANB2 can bind to lipid A, a core component of LPS, and the affinity of rZRANB2 to LPS was not inhibited by any of the sugars examined. We do not know the mechanism why Ly2.1–3 only targets Gram-negative bacteria, whereas rZRANB2 and rLy2.1–3 all can bind to LPS but not to LTA. Therefore, we speculate that is why they target Gram-negative bacteria. We hypothesize that they all act mainly on Gram-negative bacteria by initially binding to the surface LPS, followed by binding to the Gram-negative bacteria and then kill the bacteria. In addition, Sun et al. [15] have shown that CD59, another member of Ly6 family, has both bacterial-binding and inhibiting activities. The gene family exhibits several structural features typical of antimicrobial AMPs, we assume that Ly6 family is a potential source to dig for new AMPs. Based on the present information, it remains uncertain whether all the Ly6 family proteins isolated from zebrafish can function as AMPs, however, this deserves further study in the future.

The emergence of MDR microbes caused by overuse of antibiotics has caused the less efficacy of major antimicrobial drugs used in clinical settings [30] and thus demands urgent exploration of new antibiotics including AMPs. An important point about the development of antimicrobial therapeutics is that they must not be toxic to mammalian cells. We showed that rLy2.1, rLy2.2 and rLy2.3 were virtually non-cytotoxic towards human RBCs and murine RAW264.7 cells, suggesting that they have high-membrane selectivity towards bacterial cells but not mammalian cells. In summary, the present study reports the identification of three uncharacterized AMPs in the members of Ly6 superfamily in zebrafish. These AMPs can kill Gram-negative bacteria like *A. hydrophila* and *E. coli*, but are non-cytotoxic to mammalian cells. All these together indicate that Ly2.1–3 can function as AMPs.

## Abbreviations

AMP: antimicrobial peptide
*B*_max_: apparent maximum number of bindings
DAB: diaminobenzidine
DE3: *E. coli* Transetta
DMEM: Dulbecco's modified eagle medium
HRP: horseradish peroxidase
*K*_d_: equilibrium dissociation constant
LPS: lipopolysaccharide
LTA: lipoteichoic acid
Ly6: lymphocyte antigen 6
Lypd8: Ly6/PLAUR domain containing 8
Lynx1: Ly-6/neurotoxin protein 1
MDR: multidrug-resistant
NHS-LC-biotin: biotinamidohexanoic acid *N*-hydroxysuccinimide ester
PATE: prostate and testis expressed
PSCA: prostate stem cell antigen
RBC: red blood cell
rLy2.1–3: recombinant proteins Ly2.1–3
SF: superfamily
SLURP1: secreted mammalian Ly-6/uPAR-related protein 1
SLURP2: secreted mammalian Ly-6/uPAR-related protein 2
TSB: Tryptic Soy Broth
ZRANB2: zinc finger RANBP2-type containing 2
